# Supplementary figures and images for: Seasonal and periodic patterns in US COVID-19 mortality using the Variable Bandpass Periodic Block Bootstrap
Source: PLoS One. 2025 Jan 22;20(1):e0317897. doi: 10.1371/journal.pone.0317897 (PMC11753702; doi:10.1371/journal.pone.0317897)

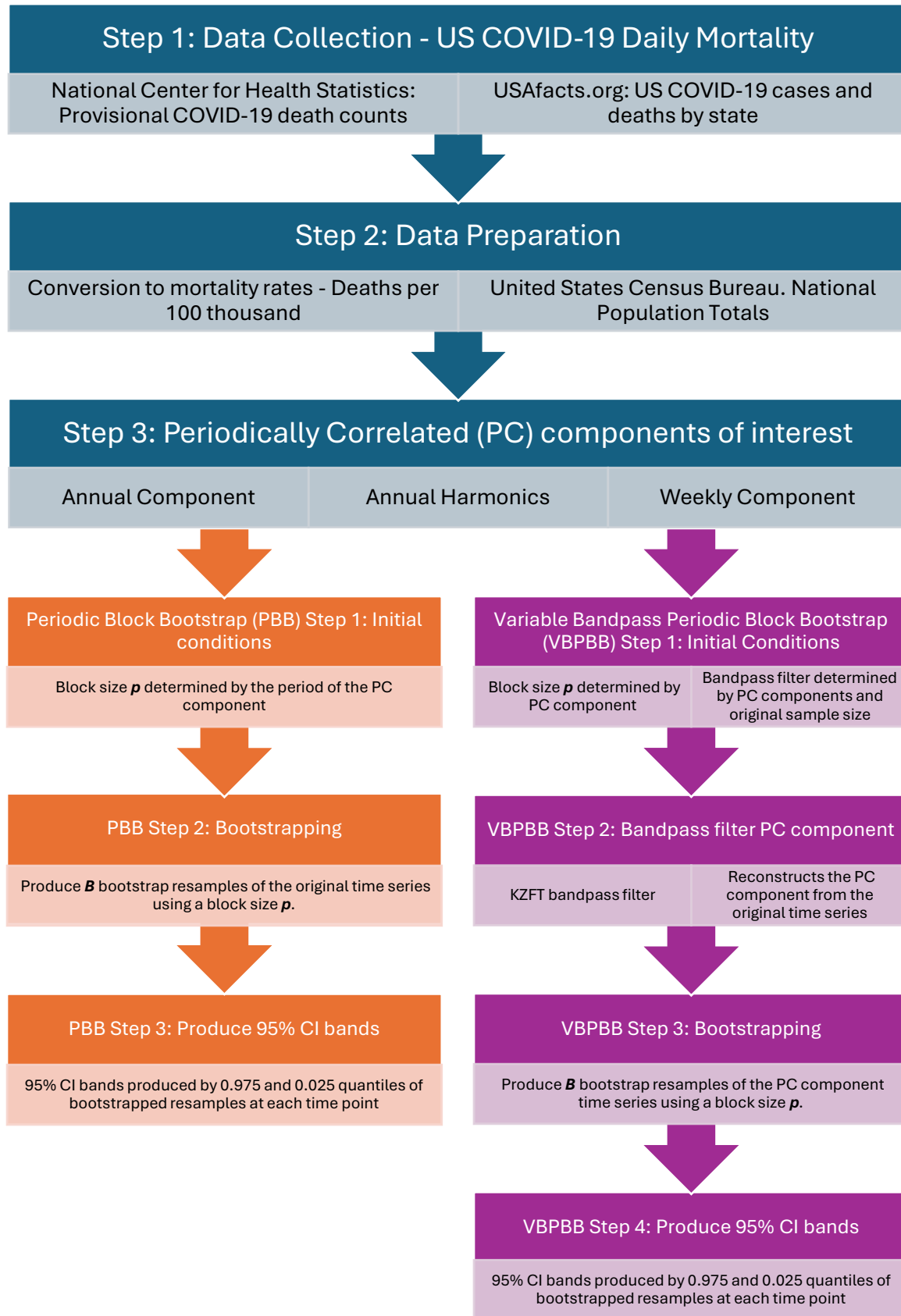

Supplement: S1 Appendix — (PDF) [file pone.0317897.s001.pdf]
